# Supplementary material for: Thermal Degradation of Photoluminescence Poly(9,9-dioctylfluorene) Solvent-Tuned Aggregate Films
Source: Polymers (Basel). 2022 Apr 15;14(8):1615. doi: 10.3390/polym14081615 (PMC9029415; doi:10.3390/polym14081615)
Supplement: Supplementary file 1 [file polymers-14-01615-s001.zip › polymers-1645366-supplementary.pdf]

Supplementary Materials

Thermal degradation of photoluminescence poly (9,9-dioctylfluorene) solvent-tuned aggregate films

Kang Wei Chew [1], Nor Azura Abdul Rahim *[1], Pei Leng Teh [1], Nurfatin Syafiqah Abdul Hisam [1] and Siti Salwa Alias [2]

[1]Faculty of Chemical Engineering Technology, Universiti Malaysia Perlis (UniMAP), Pusat Pengajian Jejawi 2, 02600 Arau, Perlis, Malaysia.
[2] Advanced Optical Materials Research Group (AOMRG), Department of Physics, Faculty of Science, Universiti Teknologi Malaysia (UTM), 81310 Skudai, Johor, Malaysia.
*Correspondence: norazura@unimap.edu.my

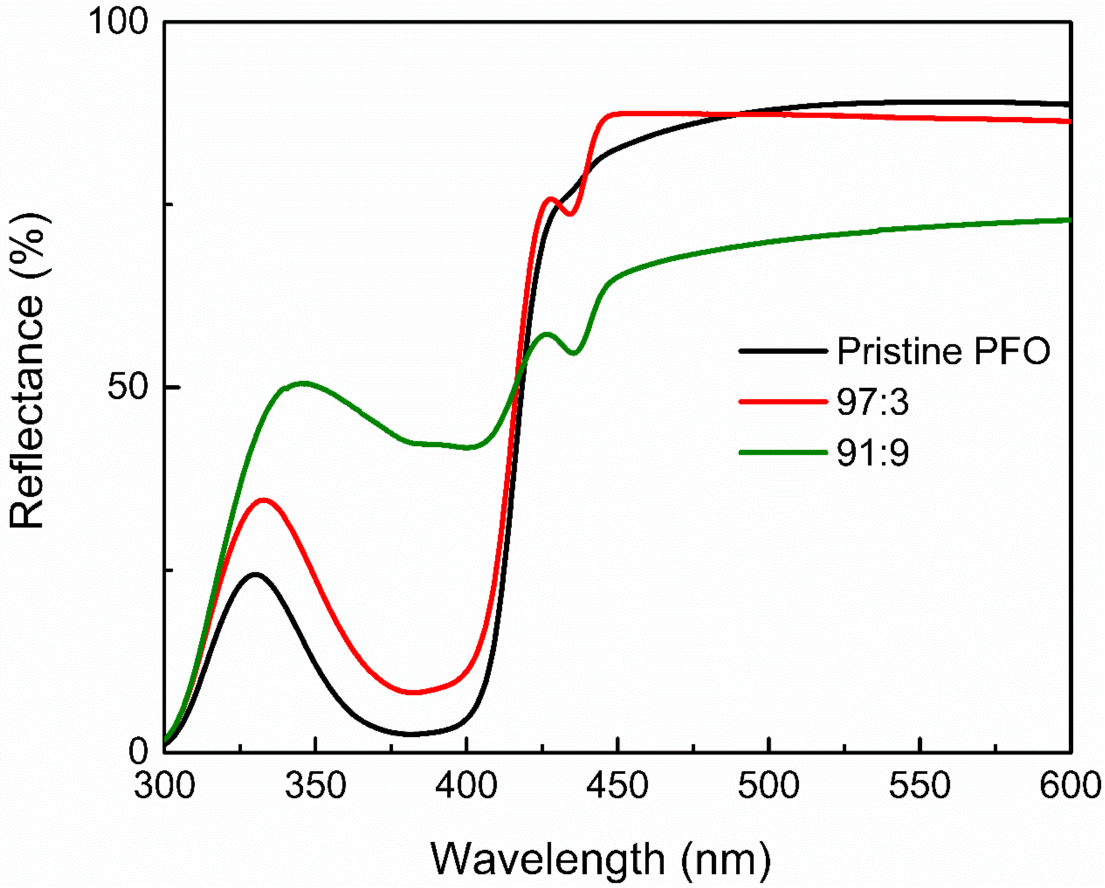

Figure S1: UV-Vis reflectance spectra of the PFO film and aggregated PFO films with 97:3 and 91:9 chloroform/methanol ratios.
